# Neutrophil-Lymphocyte Ratio in Small Renal Masses

**DOI:** 10.1155/2014/759253

**Published:** 2014-03-12

**Authors:** Wassim M. Bazzi, Sheila Z. Dejbakhsh, Melanie Bernstein, Paul Russo

**Affiliations:** ^1^Urology Service, Department of Surgery, Memorial Sloan Kettering Cancer Center, 1275 York Avenue, New York, NY 10065, USA; ^2^Columbia University Mailman School of Public Health, 722 W 168th Street, New York, NY 10032, USA; ^3^University of California San Diego School of Medicine, 9500 Gilman Drive, San Diego, CA 92093, USA

## Abstract

*Introduction*. To evaluate the association between preoperative neutrophil-lymphocyte ratio (NLR) and clinicopathologic characteristics in patients with small renal masses (SRM).* Methods*. Retrospective chart reviews of patients with renal masses ≤4 cm who underwent nephrectomy from January 2007 to July 2012 were conducted. Multivariable linear regression was used to examine the association between preoperative NLR and clinicopathologic variables.* Results*. In 1001 patients, we noted higher mean preoperative NLR in men (3.0 ± 1.4 versus 2.6 ± 1.3 in women, *P* < 0.01) and Caucasians (2.9 ± 1.4 versus 1.9 ± 0.9 in African Americans, *P* < 0.01) but no significant differences in patients with low (I-II) versus high (III-IV) American Society of Anesthesiologists (ASA) scores (2.8 ± 1.4 versus 2.9 ± 1.5, *P* = 0.18) or benign versus malignant pathology (2.9 ± 1.4 versus 2.8 ± 1.3, *P* = 0.75). Spearman correlation analysis (*ρ*) showed preoperative NLR significantly correlated with age (*ρ* = 0.15, *P* < 0.01) and preoperative serum creatinine (Crea) [*ρ* = 0.13, *P* < 0.01]. On multivariable linear regression analysis older age, male gender, Caucasian race, and preoperative Crea were predictive of higher preoperative NLR, but ASA score and tumor pathology were not.* Conclusions*. In patients with SRM, we found no association between preoperative NLR and tumor pathology.

## 1. Introduction

In 1863 Virchow proposed a relationship between inflammation and malignancy. There is now an ample body of literature on the association between chronic inflammation and cancer [1–4], with an estimated 15% of malignancies worldwide being reported as having an infectious association or etiology [5]. C-reactive protein (CRP), a serum marker for systemic inflammation, has shown good prognostication in patients with renal cell carcinoma (RCC) [6–10] and similar findings when combined with serum albumin levels in the modified Glasgow Prognostic Score [3]. However, CRP requires additional serum testing and hence is not readily available for all patients.

Neutrophil-lymphocyte ratio (NLR) is easily calculated by dividing the absolute neutrophil count by the absolute lymphocyte count from a complete blood count with differential, and its rise is a result of simultaneous increase in circulating neutrophils and decrease in lymphocytes with systemic inflammation [11]. NLR has been reported to correlate well with serum CRP levels [12] and to predict oncological outcomes in patients with RCC [12], nonclear cell RCC [13], and upper tract urothelial cell carcinoma [14].

The objective of our study was to explore for an association between preoperative NLR and age, gender, race, renal function, and tumor pathology in patients who underwent nephrectomy for SRM ≤4 cm in an effort to determine whether preoperative NLR can be incorporated in preoperative decision making.

## 2. Methods

After institutional review board approval, a retrospective chart review of patients who underwent partial or radical nephrectomy at Memorial Sloan Kettering Cancer Center from January 2007 to July 2012 was conducted (*n* = 1846). We identified 1004 patients with tumor size being ≤4 cm and stage, if malignant, classified as pT1a according to the American Joint Committee on Cancer 2010 classification [15]. Three patients were excluded for missing preoperative laboratory data. Data collected included age, gender, race, American Society of Anesthesiologists (ASA) score for medical comorbidities [16], which were divided into low (I-II) and high (III-IV), preoperative serum creatinine (Crea), preoperative estimated glomerular filtration rate (eGFR) calculated using the Chronic Kidney Disease Epidemiology Collaboration equation [17], surgical procedure and side, and final pathologic diagnosis, which were split into benign and malignant. Pretreatment laboratory complete blood count with differential, which was typically drawn within a week before surgery, was used to calculate the NLR by dividing the absolute neutrophil count by the absolute lymphocyte count. Preoperative NLR was analyzed for its association with the following variables: age, gender, race, ASA score, preoperative renal function (Crea and eGFR), and pathologic diagnoses.

### 2.1. Statistical Analysis

Preoperative and operative factors including age, gender, race, ASA score, Crea, eGFR, surgical procedure and side, and tumor pathology were described. Continuous variables were age, preoperative Crea, and preoperative eGFR, which were described by mean ± standard deviation (SD) and median with interquartile range (IQR). Categorical variables were gender, race, ASA score, surgical procedure and side, and tumor pathology, which were described by frequency and percent of total. Preoperative eGFR was described as a continuous and as a categorical variable, with the cutoff <60 mL/min/1.73 m^2^ representing chronic kidney disease (CKD) [18]. The association between preoperative factors and preoperative NLR was assessed using bivariable analysis, with continuous variables analyzed using Spearman correlation (*ρ*) and categorical variables using the Kruskal-Wallis test. Multivariable linear regression analysis was conducted to establish a model assessing the predictive value of age, gender, race, preoperative Crea, ASA score, and tumor pathology. The linear regression model was adjusted to account for heteroskedasticity. All probabilities were two sided, and a *P* < 0.05 was considered significant for all analyses. All data were analyzed using STATA 12.0 (Stata Corp., College Station, TX, USA).

## 3. Results 

Our cohort consisted of 1001 patients (Table 1). The majority (89.1%) of patients were Caucasian (*n* = 892), and 61.8% (*n* = 619) were men. Median age was 59.9 years (IQR 51.7, 68.3) and median preoperative eGFR 69.6 mL/min/1.73 m^2^ (IQR 58.7, 82.3), with 27.8% (*n* = 278) having preoperative eGFR <60 mL/min/1.73 m^2^. ASA score was high (III-IV) in 47.8% (*n* = 478) of patients. A total of 98.3% (*n* = 984) of patients underwent partial nephrectomy, and 79.8% (*n* = 799) had malignant pathology.

The results of the bivariable analysis are shown in Table 2. Men had a statistically significant higher mean preoperative NLR than women: 3.0 ± 1.4 versus 2.6 ± 1.3, respectively (*P* < 0.01). Caucasians had a higher mean preoperative NLR 2.9 ± 1.4, when compared to African Americans, 1.9 ± 0.9, and other races, 2.7 ± 1.6, (*P* < 0.01). Patients with low ASA score (I-II) did not have statistically significant different preoperative NLR when compared to patients with high ASA score (III-IV): 2.8 ± 1.4 versus 2.9 ± 1.5, respectively (*P* = 0.18). There was no significant difference in preoperative NLR between benign and malignant pathology: 2.9 ± 1.4 versus 2.8 ± 1.3, respectively (*P* = 0.75). Spearman correlation (*ρ*) analysis showed preoperative NLR had a statistically significant correlation with age (*ρ* = 0.15, *P* < 0.01), preoperative Crea (*ρ* = 0.13, *P* < 0.01), and preoperative eGFR (*ρ* = −0.13, *P* < 0.01).

A multivariable logistic linear regression model was used to establish predictive value of the variables age, gender, race, preoperative Crea, ASA score, and pathology for preoperative NLR. To preserve independence and avoid multicolinearity throughout the statistical analysis, preoperative eGFR was omitted from the model, as age, race, gender, and Crea are incorporated in eGFR calculation [17]. As shown in Table 3, older age (*P* < 0.01), male gender (*P* < 0.01), Caucasian race (*P* < 0.01), and worse preoperative Crea (*P* = 0.03) were all associated with higher preoperative NLR. ASA score and pathology had no association.

## 4. Discussion

Numerous serum inflammation markers, such as CRP [6–10], have proven to be good prognostic indicators in patients with a variety of malignancies, but they require separate laboratory testing. The easily calculated NLR, on the other hand, has shown utility as an alternative marker of systemic inflammation in critically ill patients [19], malignancies [20], and chronic medical conditions such as end-stage renal disease and diabetes [21–23].

Our study's goal was to determine whether preoperative NLR can be incorporated in preoperative decision making for patients with small renal masses, as 20% of those masses prove benign after surgery [24]. There was no difference in preoperative NLR values between benign and malignant pathologies. In addition, factors associated with higher preoperative NLR were older age, male gender, and Caucasian race though this latter finding might be spurious as 89% of our cohort was Caucasian. In terms of medical comorbidities, there was no significant difference in preoperative NLR values between patients with low or high ASA scores; however, worse preoperative renal function was associated with higher preoperative NLR. This latter association is in concordance with the medical literature and reflects the systemic inflammatory response with renal failure [21–23].

In Ohno et al. [12] report on preoperative NLR in 192 patients with nonmetastatic RCC, preoperative NLR higher than 2.7 was associated with worse recurrence-free survival after multivariable analysis with similar finding by de Martino et al. [13] when using NLR as a continuous variable in 281 patients with nonclear cell renal cell carcinoma. This is in contrast to Pichler et al. [25] finding in a large European validation study of pretreatment NLR prognostication of RCC in 678 patients, where preoperative NLR elevation was associated with worse OS but not cancer specific outcomes. Pichler et al. [25] concluded that elevated NLR reflects patients' severe cardiovascular and medical comorbidities. Contrary to those studies [12, 13, 25], we did incorporate patients' demographics and medical comorbidities in our analyses. Though this ratio appears promising for prognostication of many malignancies including genitourinary, we noted that preoperative NLR is associated with patients' demographics and renal function consistent with Pichler et al. findings [25]. We believe our findings must be taken into consideration for careful interpretation of NLR especially in RCC prognostication, and NLR lacks utility in clinical practice when selecting management options for small renal masses.

Our study is limited by its retrospective nature and as alluded to by Pichler et al. [25] this marker is not specific and can be influenced by factors that we did not control for in this study such as active infection, hematologic and inflammatory diseases, and stress at time of blood draw.

## 5. Conclusions

In our review of patients with small renal masses, we found no association between preoperative NLR and tumor pathology. Older age, male gender, and preoperative serum Crea were associated with higher NLR.

## Figures and Tables

**Table 1:** Baseline characteristics for 1001 patients with renal masses ≤4 cm. Values are expressed as mean (SD), median (IQR), or frequency (percent).

Age at surgery, median (IQR)	59.9 (51.7, 68.3)
Gender	
Male (%)	619 (61.8%)
Female (%)	382 (38.2%)
Race	
Caucasian (%)	892 (89.1%)
African American (%)	47 (4.7%)
Other (%)	62 (6.2%)
ASA score	
I-II (%)	523 (52.2%)
III-IV (%)	478 (47.8%)
Preoperative Crea, median	1.1 (0.9, 1.2)
Preoperative eGFR mL/min/1.73 m^2^, median	69.6 (58.7, 82.3)
<60	278 (27.8%)
≥60	723 (72.2%)
Surgical procedure	
Partial (%)	984 (98.3%)
Radical (%)	17 (1.7%)
Side of procedure	
Right (%)	492 (49.2%)
Left (%)	509 (50.8%)
Pathology	
** **Benign (%)	**202 (20.2%)**
** **Oncocytoma (%)	149 (14.9%)
** **Angiomyolipoma (%)	31 (3.1%)
** **Other benign (%)	22 (2.2%)
** **Malignant (%)	**799 (79.8%)**
** **Conventional RCC (%)	538 (53.7%)
** **Papillary RCC (%)	118 (11.8%)
** **Chromophobe RCC (%)	77 (7.7%)
** **Cystic RCC (%)	43 (4.3%)
** **Unclassified RCC (%)	11 (1.1%)
** **Other malignant (%)	12 (1.2%)

**Table:** (a)

Characteristic	Mean preoperative NLR (SD)	*P* value
Age	2.8 (1.4)	**<**0.01^a^
Gender		**<**0.01^b^
Male	3.0 (1.4)	
Female	2.6 (1.3)	
Race		**<**0.01^b^
Caucasian	2.9 (1.4)	
African American	1.9 (0.9)	
Other	2.7 (1.6)	
ASA score		0.18^b^
I-II	2.8 (1.4)	
III-IV	2.9 (1.5)	
Preoperative Crea	2.8 (1.4)	**<**0.01^a^
Preoperative eGFR	2.8 (1.4)	**<**0.01^a^
Pathology		0.75^b^
Benign	2.9 (1.4)	
Malignant	2.8 (1.3)	
Pathological subtypes		0.34^b^
Benign		
Oncocytoma	2.9 (1.4)	
Angiomyolipoma	2.8 (1.4)	
Other benign	2.7 (1.2)	
Malignant		
Conventional RCC	2.8 (1.3)	
Papillary RCC	3.1 (1.7)	
Chromophobe RCC	2.5 (1.1)	
Cystic RCC	3.1 (2.3)	
Unclassified RCC	2.5 (1.3)	
Other malignant	2.3 (0.9)	

^a^Measure of association by Spearman correlation.

^b^Measure of association by Kruskal-Wallis test.

**Table:** (b)

	*ρ*	*P* value
Age	0.15	**<**0.01
Preoperative Crea	0.13	**<**0.01
Preoperative eGFR	−0.13	**<**0.01

**Table 3:** Multivariable linear regression analysis for predicting preoperative NLR.

	Beta coefficient	95% Confidence interval	*P* value
Age	0.01	0.005, 0.020	**<**0.01
Gender			
Male	Reference		
Female	−0.28	−0.47, −0.10	**<**0.01
Race			
Caucasian	Reference		
African American	−0.95	−1.23, −0.67	**<**0.01
Other	−0.21	−0.61, 0.19	0.30
Preoperative Crea	0.40	0.03, 0.76	0.03
ASA score			
I-II	Reference		
III-IV	0.07	−0.10, 0.24	0.42
Pathology			
Benign	Reference		
Malignant	0.01	−0.20, 0.22	0.94
